# Chlorantraniliprole Resistance and Associated Fitness Costs in Fall Armyworm (*Spodoptera frugiperda*): Implications for Resistance Management

**DOI:** 10.3390/insects16121232

**Published:** 2025-12-06

**Authors:** Arzlan Abbas, Faisal Hafeez, Ali Hasnain, Ayesha Iftikhar, Muhammad Hassan Khan, Farman Ullah, Ahmed M. M. Elkady, Chen Ri Zhao, Xiaohe Sun

**Affiliations:** 1 College of Plant Protection, Jilin Agricultural University, Changchun 130118, China; abbasarzlan6@gmail.com (A.A.); chenrizhao@jlau.edu.cn (C.R.Z.); 2 Department of Entomology, University of Agriculture, Faisalabad 38040, Punjab, Pakistan; 3 Entomological Research Institute, Ayub Agricultural Research Institute, Faisalabad 38850, Punjab, Pakistan; faisalhafeez143@gmail.com (F.H.);; 4 College of Plant Protection, Nanjing Agricultural University, Nanjing 211800, China; alihasnain88@outlook.com; 5 Institute of Bio-Interaction, Xianghu Laboratory, Hangzhou 311258, China; 6 Faculty of Law & Political Sciences, King Saud University, Riyadh 11451, Saudi Arabia; 7 College of Information Technology, Jilin Agricultural University, Changchun 130118, China

**Keywords:** chlorantraniliprole resistance, fitness cost, integrated pest management, life table analysis, realized heritability, resistance management, *Spodoptera frugiperda*

## Abstract

*Spodoptera frugiperda* J.E. Smith (Lepidoptera: Noctuidae) represents a major threat to maize production across Pakistan, with chemical control serving as the predominant management approach. The intensive application of insecticides, particularly diamide compounds such as chlorantraniliprole, has escalated concerns regarding resistance evolution in field populations. This study evaluated the insecticidal efficacy of seven commonly used compounds against geographically diverse field-collected populations of *S. frugiperda* from major maize-growing regions of Pakistan, revealing significant inter-population variability in susceptibility profiles. Chlorantraniliprole was selected for comprehensive transgenerational screening based on moderate baseline LC_50_ values and optimal laboratory colony establishment parameters. A representative field strain underwent six consecutive generations of selection pressure at LC_70_ concentrations, resulting in a 4.48-fold increase in resistance levels with a realized heritability (*h*^2^) of 0.198. Predictive modeling using established quantitative genetic frameworks demonstrated that resistance evolution rates are critically dependent on both selection intensity and genetic parameters. Under constant *h*^2^ = 0.198, increasing selection intensity substantially accelerated resistance development, with 10-fold resistance achievable in approximately 18 generations at 80% selection intensity (slope = 2.696) compared to 36 generations at lower intensities (slope = 4.696). Sensitivity analysis revealed that heritability variations from 0.148 to 0.248 could reduce generation requirements from >40 to ~25 generations when slope was maintained at 3.696. Life table analyses of the chlorantraniliprole-selected strain demonstrated significant fitness costs manifested as extended developmental periods, reduced reproductive output, and decreased intrinsic rate of population increase (*r*), indicating evolutionary trade-offs associated with resistance acquisition. These findings provide crucial insights for developing sustainable management strategies, highlighting the importance of integrating resistance monitoring, refuge-based approaches, and rotation with insecticides of different modes of action to delay resistance buildup in field populations. Such data-driven management frameworks are vital for maintaining the long-term efficacy of diamides in Pakistan’s maize production systems.

## Simple Summary

The fall armyworm, *Spodoptera frugiperda*, is a highly destructive invasive pest that affects a variety of crops, especially maize, rice, and forage crops, causing significant economic losses and threatening sustainable agriculture worldwide. This study evaluates the insecticidal susceptibility of multiple field-collected *S. frugiperda* populations from Punjab, Pakistan, providing updated insights into their current resistance status. One population was subjected to selection pressure with chlorantraniliprole, a commonly used diamide insecticide in Pakistan. Through transgenerational screening, the study compared the life history traits and relative fitness of the chlorantraniliprole-selected strain with a laboratory-maintained susceptible strain. The findings reveal the extent of resistance development and highlight the associated fitness costs that may influence the long-term stability and spread of resistance in field populations. Overall, this research enhances understanding of resistance dynamics in *S. frugiperda* and supports the development of more effective and sustainable insecticide resistance management strategies.

## 1. Introduction

*Spodoptera frugiperda* is a highly destructive pest causing major economic losses in maize and other cereal crops worldwide. It is distinguished by its exceptional reproductive potential, absence of diapause, wide host range, strong dispersal ability and rapid adaptation to new environments enabling it to infest over 353 species across 76 plant families [1,2,3]. Originally endemic to the tropical and subtropical regions of the Americas, *S. frugiperda* has rapidly expanded its range to Africa and Asia [2,3,4,5], posing a major threat to agricultural production. Its presence in China since 2018 and subsequent spread to Pakistan has led to substantial yield losses, with reductions in maize production reaching up to 60%, highlighting its global importance for food security [6]. Despite biological control options [7,8,9], chemical insecticides have been widely used against *S. frugiperda*. However, the repeated application of these products has resulted in non-target effects on beneficial insects as well as resistance development in targeted pests [10,11,12,13], complicating control efforts.

Diamide insecticides, such as chlorantraniliprole, have been integral to integrated pest management (IPM) strategies for controlling *S. frugiperda* and other Lepidoptera species. These insecticides function by targeting ryanodine receptors (RyRs) within the endoplasmic reticulum of insects, thereby disrupting calcium ion release and inducing irreversible muscle contraction and paralysis [14,15,16]. Diamides are well organized for their low mammalian toxicity and relatively high environmental safety [15] compared to conventional chemistries. Nevertheless, recent reports of resistance development in *S. frugiperda*, have raised serious concerns regarding their sustained effectiveness in IPM programs [17,18,19,20]. Similar sublethal and transgenerational effects have previously been documented for neonicotinoids, highlighting how sublethal exposures can influence insect physiology and population dynamics [21], underscoring the need for research on these effects to better understand their broader ecological and evolutionary implications.

Transgenerational screening studies have revealed significant insights into the realized heritability (*h*^2^) of insecticide resistance in *S. frugiperda*, providing crucial parameters for predicting resistance evolution rates. Recent investigations on lambda-cyhalothrin resistance demonstrated a realized heritability (*h*^2^) of 0.11 after 21 generations of continuous selection pressure, indicating a moderate capacity for resistance trait inheritance [22]. Similarly, studies on diamide insecticides have shown varying heritability estimates depending on the specific active ingredient and selection intensity. The inheritance patterns typically follow autosomal modes with varying degrees of dominance, where chlorantraniliprole resistance was characterized as autosomal, incompletely recessive and monogenic [23]. Fluxametamide resistance risk assessment revealed rapid development potential under laboratory selection conditions, with significant resistance ratios emerging within 10 generations [24]. These heritability estimates are critical for resistance management programs, as they enable prediction of resistance spread rates and inform decisions regarding insecticide rotation strategies and refuge requirements. Moderate to high heritability values across different insecticide classes suggest that *S. frugiperda* populations possess substantial genetic variation for resistance traits, making them particularly prone to rapid adaptation under sustained selection pressure [25].

Fitness cost analyses through transgenerational screening have revealed complex relationships between insecticide resistance and biological performance in *S. frugiperda*, with costs varying significantly based on the resistance mechanism, insecticide class, and environmental conditions. Moreover, insect feeding responses and their interactions with plant defense systems [26,27], can influence resistance-associated fitness outcomes, providing essential ecological context for interpreting performance variation. Chlorpyrifos resistance studies demonstrated measurable fitness costs on different host plants, with resistant populations showing reduced developmental rates and fecundity parameters compared to susceptible strains [28]. Temperature-dependent fitness costs have been particularly well-documented for chlorantraniliprole and spinetoram resistance, where resistant strains exhibited greater fitness penalties under suboptimal temperature conditions [18]. Diamide-resistant strains demonstrated host plant-dependent fitness costs, with greater penalties observed on certain crop species compared to others [29]. These findings indicate that fitness costs in *S. frugiperda* are not universal but rather depend on the specific resistance alleles, their interactions with genetic background, and environmental factors. Comparable selection-induced fitness costs have also been demonstrated in other pests, such as *Tuta absoluta*, *Chilo suppressalis*, and *Aphis gossypii* under selection pressure, suggesting that resistance often carries biological trade-offs [30,31,32]. The variable nature of these costs has important implications for resistance management, as populations with minimal fitness costs may maintain resistance alleles even in the absence of selection pressure, potentially reducing the effectiveness of resistance mitigation strategies based on fitness cost exploitation [29].

Although various insecticides are used to manage *S. frugiperda* in Pakistan, comprehensive data on their efficacy against field populations remain limited. Among these, chlorantraniliprole has shown potential but lacks systematic evaluation for resistance development. This study aimed to assess the insecticidal toxicity of several compounds across different field populations, with chlorantraniliprole selected for transgenerational screening over six successive generations. Based on this selection, the realized heritability (*h*^2^) of resistance was estimated, along with the projected number of generations required for a 10-fold resistance increase. Additionally, comparative life table analyses were conducted between resistant and susceptible strains to assess fitness costs, using the age-stage, two-sex life table approach, which provides an integrated evaluation of survival, development, and reproduction by considering both sexes and individual variability for more accurate insights into population dynamics [33,34,35,36,37]. Overall, this research enhances understanding of resistance dynamics and supports effective resistance monitoring and management strategies for *S. frugiperda*.

## 2. Materials and Methods

### 2.1. Insects

**Figure 1:**
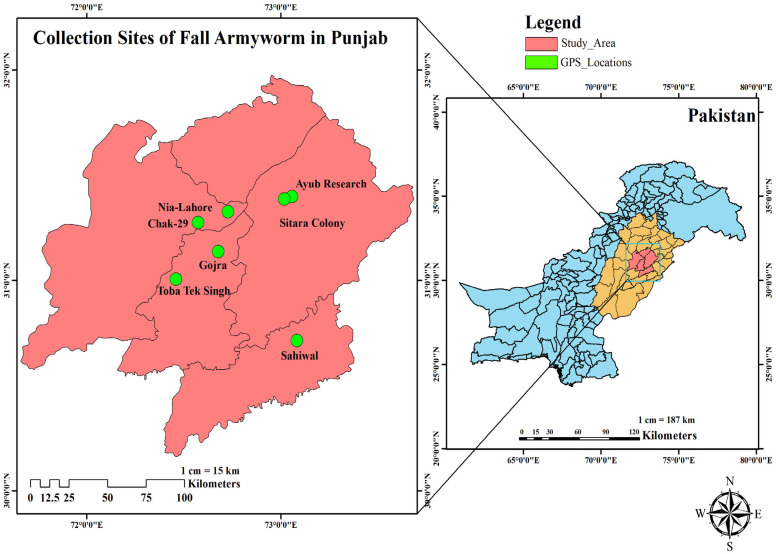
Collection sites of fall armyworm in Punjab, Pakistan.

Field populations of *S. frugiperda* were collected from major maize-growing regions across Punjab, Pakistan, during the peak infestation period (July–September 2024). The collection sites included Chak-29 (CT: 31.273952° N, 72.571701° E), Sitara-Colony (SC: 31.386235° N, 73.016011° E), Ayub-Research (AR: 31.396910° N, 73.056505° E), Gojra (GJ: 31.136026° N, 72.676201° E), Nia-Lahore (NL: 31.325444° N, 72.726408° E), Toba-Tek-Singh (TTS: 31.004400° N, 72.458250° E), and Sahiwal (SHW: 30.711656° N, 73.080906° E). A map of collection sites (Figure 1) illustrates their geographic positions and relative distances. Systematic sampling was conducted in 2–3 fields per location, where every 5th row was inspected and 10 plants per row examined, covering 50–60 plants per field. Approximately 200 third- to fourth-instar larvae were pooled from each location for laboratory colony establishment. The sites were characterized based on general cropping history, observed pest pressure, reported insecticide use, and local management practices, providing a representative sample of agroecological variation in the region.

Field collected larvae were transported to the Insect Rearing Laboratory (Entomological Research Institute, Faisalabad) in ventilated containers with fresh maize leaves collected from pesticide-free plants. Larvae were maintained under controlled conditions (26 ± 1 °C, 65 ± 5% RH, 14:10 h L:D photoperiod). Adult moths were reared in oviposition cages (30 cm diameter × 30 cm height) each containing 25 pairs of male and females. Cages were lined with paper towels to facilitate egg deposition, and oviposition substrates were replaced daily. Adults were provided with a 10% honey solution via cotton wicks as a carbohydrate source. Egg masses were collected daily, surface-sterilized with 2% sodium hypochlorite solution, and incubated until hatching. Neonate larvae were transferred to plastic boxes containing pesticide-free fresh maize leaves, which were replaced every 24 h, to prevent cannibalism and ensure adequate nutrition.

### 2.2. Insecticides

Commercial formulations of insecticides were procured from authorized dealers and stored according to manufacturer specifications. The test insecticides included bistrifluron (Hanaro^®^, 10EC, Jafar Brothers, active ingredient 100 mL/acre, emulsifiable concentrate formulation), chlorantraniliprole (Coragen^®^ 200SC, DuPont, active ingredient 100 mL/acre, suspension concentrate formulation), flubendiamide (Belt^®^, 480SC, Bayer CropScience, active ingredient 50 mL/acre, suspension concentrate formulation), lufenuron (Match^®^, 050EC, Syngenta Pakistan Ltd., active ingredient 240 mL/acre, emulsifiable concentrate formulation), emamectin benzoate (Proclaim^®^, 1.9EC, Syngenta Pakistan Ltd., active ingredient 240 mL/acre, emulsifiable concentrate formulation), indoxacarb (Steward^®^ 150SC, DuPont, active ingredient 175 mL/acre, suspension concentrate formulation) and cyantraniliprole (Benevia^®^, 100OD, FMC PVT Ltd., active ingredient 200 mL/acre, oil dispersion formulation). The stock solutions (1000 ppm) were prepared in distilled water with 0.1% Triton X-100 as surfactant and stored at 4 °C. Working concentrations were prepared fresh for each bioassay through serial dilution of stock solutions.

### 2.3. Bioassays

Insecticide susceptibility was determined using standardized leaf-dip bioassays following modified protocols from previous studies [11,38,39]. Fresh maize leaves (V6–V8 growth stage) were collected from pesticide-free plants and cut into uniform discs (8 cm diameter). Leaf discs were dipped for 10s in test concentrations, air-dried for 30 min under ambient conditions, and placed in plastic Petri dishes (9 cm diameter) lined with moistened filter paper.

Preliminary range-finding bioassays were conducted to determine five appropriate concentration ranges for definitive dose–response studies. The final concentration ranges tested were: bistrifluron: 0.1, 1.0, 5.0, 10.0, 20.0 mg/L, chlorantraniliprole: 0.1, 1.0, 5.0, 10.0, 15.0 mg/L, flubendiamide: 0.2, 1.0, 5.0, 15.0, 25.0 mg/L, lufenuron: 0.2, 5.0, 10.0, 15.0, 20.0 mg/L, emamectin benzoate: 0.05, 0.5, 1.0, 5.0, 10.0 mg/L, indoxacarb: 1.0, 5.0, 10.0, 15.0, 25.0 mg/L and cyantraniliprole: 0.1, 1.0, 5.0, 10.0, 15.0 mg/L, respectively.

Third instar larvae (48 ± 6 h old) were used for all bioassays to minimize developmental variation. Twelve larvae were placed individually in the wells of 12-well compartment plates containing treated leaf discs, with three replicates per concentration (n = 36 larvae per concentration). Control treatments consisted of leaf discs dipped in distilled water with surfactant and control mortality was monitored in each assay. Bioassays with control mortality exceeding 10% were repeated, while recorded mortalities from assays with control mortality were corrected using Abbott’s formula [40]. Bioassay plates were sealed with parafilm to avoid larval escape and incubated under the same environmental conditions as colony maintenance. Larval mortality was assessed after 48 h of exposure, with larvae considered dead if they failed to respond to gentle prodding with a fine brush. Larvae exhibiting pronounced signs of intoxication, such as impaired movement, paralysis, or severe lethargy, were considered dead.

### 2.4. Transgenerational Screening and Fitness Cost

One representative field population showing moderate resistance levels was selected for transgenerational screening studies. Chlorantraniliprole was chosen for subsequent experiments due to its widespread use in Pakistani maize fields, consistent baseline lethal concentration (LC) values, and ability to support stable colonies under continuous exposure, critical for multi-generational studies. Its frequent application and emerging resistance risks make it a key candidate for resistance monitoring and management. The population was subjected to continuous selection pressure for six consecutive generations (F_1_–F_6_) using LC_70_ concentrations of chlorantraniliprole as the selection agent. Each generation, approximately 500 third-instar larvae were exposed to the predetermined LC_70_ concentration, with survivors allowed to complete development, mate, and reproduce. LC_70_ values were recalculated every second generation to maintain consistent selection pressure as resistance levels increased. A parallel susceptible population was maintained without insecticide exposure to serve as a control for fitness comparisons.

Life-table studies were conducted to quantify fitness costs associated with resistance development in the selected population. One hundred newly hatched larvae from both resistant (F_6_) and susceptible control populations were randomly selected and reared individually under identical conditions. Daily observations were recorded for survival, developmental duration, molting success, pupal weight, adult emergence, longevity, mating success, and reproductive output. Female fecundity was assessed by counting total egg production over the oviposition period, while egg viability was determined through hatch rate evaluations. Life table parameters including survival rate (*l_x_*), age-specific fecundity (*m_x_*), net reproductive rate (*R*_0_), intrinsic rate of increase (*r*), finite rate of increase (*λ*), and generation time (*T*) were calculated using the age-stage, two-sex life table approach implemented in TWOSEX-MS chart software (version 2023.03.26).

### 2.5. Realized Heritability

Realized heritability (*h*^2^) of chlorantraniliprole resistance was calculated as described by Tabashnik et al. [41] and Hafez et al. [42]:

*h*^2^ = *R*/*S*,

where *h*^2^ is the realized heritability, *R* represents the response to selection and *S* represents the selection differential.

*R* = log (Final LC_50_ − Initial LC_50_)/*n*

where *n* represents the number of generations, Final LC_50_ refers to the final generation (F_6_) while initial LC_50_ for initial generation.

*S* was estimated as:

*S* = *i* × *σp*

where *i* represents the selection mortality, calculated following the method described by Tabashnik and McGaughey [43]:

*i* = 1.583 − 0.0193336*p* + 0.000048*p*^2^ + 3.65194/*p*

where *p* is the survival percentage of the selected strain (F_0_–F_6_) when screened with chlorantraniliprole.

The term *σp* was obtained as:

*σp* = 1/average slope (F_0_–F_6_)

The number of generations (G) required to achieve 10-fold resistance was predicted using the following formula:

G = 1/R

Different selection intensities were considered for giving more comprehensive trend of resistance development. These predictions provide insights into the potential rate of resistance evolution under field conditions and inform resistance management strategies.

### 2.6. Statistical Analysis

Concentration-mortality data from bioassays were analyzed using probit analysis in SPSS v.17.0 software (SPSS Inc., Chicago, IL, USA). Median lethal concentrations (LC_50_) and their 95% fiducial limits were calculated for each population against different insecticides. Resistance ratios (RR) were computed by dividing the LC_50_ values of subsequent generations by the LC_50_ value of the first screening generation (F_0_).

Life table data were analyzed using the age-stage, two-sex life table theory [33,36,44,45] implemented in TWOSEX-MSChart software (version 2023.03.26). The software estimated age-stage specific survival rate (*s_xj_*), age-specific survival rate (*l_x_*), female age-specific fecundity (*f_x_*_9_), age-specific fecundity of the total population (*m_x_*), age-specific net maternity (*l_x_m_x_*), age-stage specific life expectancy (*e_xj_*), and age-stage specific reproductive value (*v_xj_*) [46,47]. Individual life table parameters were compared using Tukey’s HSD test after appropriate transformation to meet normality assumptions. Population parameters (*R*_0_, *r*, *λ*, *T*) were estimated between resistant and susceptible populations using paired bootstrap tests (100,000 iterations) to determine statistical significance [48,49,50,51,52]. All graphs and figures were prepared using Sigma Plot software (Version 14.0, Systat Software Inc., San Jose, CA, USA). Life table parameters were presented as mean ± standard error with appropriate statistical annotations.

## 3. Results

### 3.1. Insecticidal Toxicity of Various Insecticides Against Field Populations of S. frugiperda

The estimated LC_50_ values for bistrifluron ranged from 1.238 to 3.923 mg/L across the seven populations tested. The highest LC_50_ was observed in CT (3.923 mg/L), whereas the lowest was in SHW (1.238 mg/L). Slopes of the regression lines varied moderately (1.143–1.751), indicating differences in response homogeneity. Chlorantraniliprole exhibited relatively uniform toxicity, with LC_50_ values between 0.983 and 1.490 mg/L. The lowest LC_50_ was recorded in TTS (0.983 mg/L), indicating high susceptibility, while NL showed slightly elevated tolerance (1.490 mg/L). The LC_50_ values for flubendiamide varied from 0.919 to 4.678 mg/L, with TTS exhibiting the highest tolerance and SHW the highest susceptibility (Table 1).

Lufenuron toxicity varied considerably, with LC_50_ values spanning 2.087 to 7.973 mg/L. The SC population showed the highest tolerance, while TTS was the most susceptible. Emamectin benzoate demonstrated strong efficacy across all populations, with LC_50_ values ranging narrowly between 0.166 and 0.553 mg/L. The TTS population was the most susceptible, while SC had the highest LC_50_. LC_50_ values of Indoxacarb ranged from 3.493 to 5.044 mg/L, indicating moderate toxicity across populations. The highest LC50 was observed in SHW (5.044 mg/L), while the lowest occurred in CT (3.493 mg/L). Cyantraniliprole exhibited high toxicity with LC_50_ values ranging from 0.415 to 1.126 mg/L. AR was the most susceptible population, whereas SC had the highest LC_50_ (Table 1).

### 3.2. Transgenerational Selection for Chlorantraniliprole Resistance

The field population of *S. frugiperda* from NL was subjected to continuous selection with chlorantraniliprole at the LC_70_ level across six successive generations (F_0_–F_6_). A steady increase in LC_50_ values was observed, rising from 1.217 mg/L in the parental generation (F_0_) to 5.452 mg/L in F_6_. Correspondingly, the resistance ratio (RR) increased from 1.0 to 4.48, indicating a progressive development of resistance over generations (Table 2). These results confirm that sustained selection pressure can significantly enhance chlorantraniliprole resistance in *S. frugiperda*, highlighting the importance of proactive resistance management strategies in the field.

### 3.3. Realized Heritability of Chlorantraniliprole Resistance

The realized heritability (*h*^2^) of resistance to chlorantraniliprole in *S. frugiperda* was estimated at 0.198 following six generations of selection. The LC_50_ increased from 1.217 mg/L in the parental generation (F_0_) to 5.452 mg/L in the F_6_ generation, indicating a notable selection response (*R* = 0.109). The selection differential (*S*) was calculated as 0.549, with a mean phenotypic variance (*σ_P_*) of 0.271. The moderate heritability value suggests that resistance development is genetically based and may progress under continued selection pressure (Table 3). This finding underscores the potential for chlorantraniliprole resistance to evolve in field populations if not managed properly.

**Figure 2:**
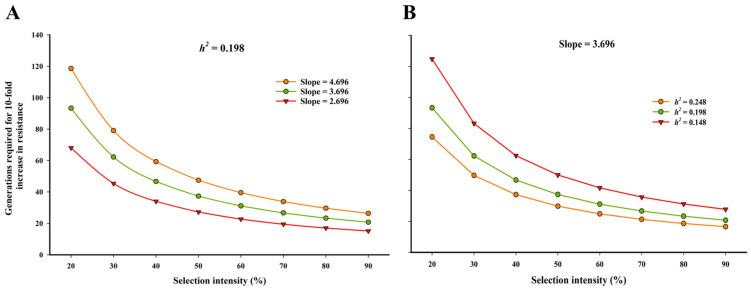
Estimated number of generations required for a 10-fold increase in chlorantraniliprole resistance in *S. frugiperda* under varying selection intensities. (**A**) Influence of different slope values (2.696, 3.696, and 4.696) with constant realized heritability (*h*^2^ = 0.198). (**B**) Influence of different realized heritability values (*h*^2^ = 0.148, 0.198, and 0.248) with a fixed slope (3.696).

To predict the long-term risk of chlorantraniliprole resistance in *S. frugiperda*, we modeled the estimated number of generations required to achieve a 10-fold increase in resistance under different selection intensities. As shown in Figure 2, under a constant heritability (*h*^2^ = 0.198), the number of generations decreased significantly with increasing selection intensity, particularly at lower slope values (Figure 2A). At 80% selection intensity, resistance could evolve in ~18 generations when the slope was 2.696, while it would take ~36 generations at a slope of 4.696. Similarly, with a fixed slope of 3.696 (Figure 2B), increased heritability sharply reduced the number of generations required for resistance development—from over 40 generations at *h*^2^ = 0.148 to approximately 25 generations at *h*^2^ = 0.248. These simulations indicate that both realized heritability and slope parameters critically influence the pace of resistance evolution.

### 3.4. Life Table Parameters for Developmental Stages

The life table analysis revealed significant differences in several developmental and reproductive traits between the chlorantraniliprole-selected strain (Sfr-Sel-F_6_) and the unselected laboratory strain (Sfr-WT), indicating a fitness cost associated with resistance development. Egg and early larval instars (1st–5th) showed no significant differences between the two strains, suggesting that chlorantraniliprole resistance did not affect the early developmental stages. However, the 2nd instar duration was slightly but significantly prolonged in the Sfr-Sel-F_6_ strain (*p* = 0.048), and a more marked extension was observed in the 6th instar (*p* < 0.001), where larvae from the resistant strain took significantly longer to develop than those from the susceptible strain (Table 4).

Consequently, the total larval duration was significantly longer in the selected strain (17.05 days vs. 16.54 days; *p* = 0.006), indicating slower larval development as a potential cost of resistance. Both male and female pupal durations were significantly extended in the Sfr-Sel-F6 strain (*p* = 0.001 and 0.016, respectively), further confirming delayed development. Most notably, adult duration was significantly reduced in both sexes of the resistant strain. Males lived 9.35 days versus 12.36 days in the susceptible strain (*p* < 0.001), and females lived 9.85 days compared to 12.87 days (*p* < 0.001). This substantial reduction in adult lifespan may negatively affect reproductive potential and overall fitness (Table 4).

### 3.5. Adult Life Table Parameters

The comparative analysis of life table parameters between the chlorantraniliprole-selected strain (Sfr-Sel-F6) and unselected laboratory strain (Sfr-WT) of *S. frugiperda* revealed significant fitness costs associated with resistance development. Although male longevity showed no significant difference between the two strains (42.11 days in Sfr-WT vs. 40.60 days in Sfr-Sel-F_6_; *p* = 0.073), female adults of the resistant strain exhibited significantly reduced longevity (41.46 days) compared to the unselected strain (43.51 days; *p* = 0.002). The adult pre-oviposition period (APOP) remained statistically similar between the strains, suggesting that initial reproductive readiness was unaffected. However, the total pre-oviposition period (TPOP) was significantly extended in the resistant strain (34.44 days) relative to the susceptible strain (33.52 days; *p* = 0.016), likely due to prolonged larval or pupal development stages (Table 5).

Reproductive output was notably impaired in the chlorantraniliprole-selected strain. Females from Sfr-Sel-F_6_ laid significantly fewer eggs (1153.17 eggs/female) than those from Sfr-WT (1486.48 eggs/female; *p* < 0.001), indicating a strong reduction in fecundity. Additionally, the oviposition duration was shorter in the resistant strain (5.14 days) compared to the unselected strain (5.41 days; *p* = 0.015). These results collectively suggest that while chlorantraniliprole resistance enhances insect survival under chemical pressure, it imposes measurable trade-offs in terms of reduced female longevity, delayed reproductive timing, and diminished fecundity. Such fitness costs may limit the long-term persistence and spread of resistance alleles in the absence of selection pressure (Table 5).

### 3.6. Fitness Cost and Population Parameters

The demographic comparison between the chlorantraniliprole-selected strain (Sfr-Sel-F_6_) and the unselected laboratory strain (Sfr-WT) of *S. frugiperda* revealed significant reductions in key population growth parameters associated with resistance development. The intrinsic rate of increase (*r*) was significantly lower in the resistant strain (0.162 d^−1^) compared to the susceptible strain (0.179 d^−1^; *p* = 0.002), indicating a reduced potential for population expansion under favorable conditions. Similarly, the finite rate of increase (*λ*) decreased significantly from 1.196 d^−1^ in Sfr-WT to 1.176 d^−1^ in Sfr-Sel-F_6_ (*p* = 0.002), further confirming the reduced reproductive efficiency of the resistant population (Table 6).

The net reproductive rate (*R*_0_), which reflects the average number of female offspring produced per female, also declined markedly in the resistant strain (415.517 offspring/individual) compared to the susceptible strain (684.116 offspring/individual; *p* = 0.005). Although the mean generation time (*T*) was slightly longer in Sfr-Sel-F_6_ (37.068 d) than in Sfr-WT (36.479 d), the difference was not statistically significant (*p* = 0.112), suggesting only a marginal delay in generation turnover due to resistance. Additionally, the female ratio (*Nf/N*) did not differ significantly between strains (*p* = 0.430), indicating that sex ratios remained relatively stable despite selection pressure. Notably, the relative fitness (*Rf*) of the resistant strain was calculated at 0.61, suggesting a 39% reduction in overall fitness compared to the susceptible strain. These findings collectively demonstrate that while resistance to chlorantraniliprole may improve survival under insecticide exposure, it imposes substantial fitness costs that can hinder population growth and reproductive success in untreated environments (Table 6).

**Figure 3:**
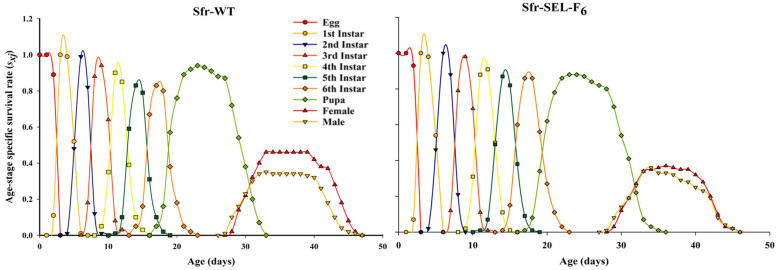
Age-stage specific survival rate (*s_xj_*) for the laboratory strain (Sfr-WT) and chlorantraniliprole-selected strain (Sfr-SEL-F_6_) of *S. frugiperda* against chlorantraniliprole.

The age-stage specific survival rate (*s_xj_*) of *S. frugiperda* for both the laboratory strain (Sfr-WT) and the chlorantraniliprole-selected strain (Sfr-SEL-F_6_). In both strains, the overlapping curves reflect the stage differentiation and variability in development time. However, compared to Sfr-WT, the Sfr-Sel-F_6_ strain exhibited slightly prolonged durations across several larval stages—most notably the 6th instar—and showed delayed pupation and adult emergence. Additionally, a slight reduction in the survival rates of pupae and adults is evident in the Sfr-Sel-F6 strain. These differences suggest that selection with chlorantraniliprole may affect developmental timing and reduce survivorship at later stages, indicative of a fitness cost associated with resistance (Figure 3).

**Figure 4:**
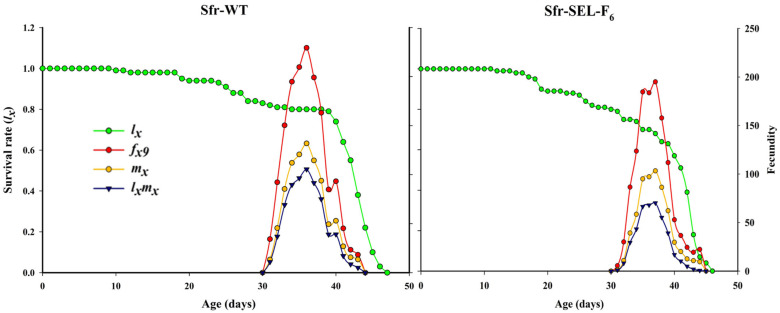
The survival rate (*l_x_*), female age-specific fecundity (*f_x_*_9_), age-specific fecundity of total population (*m_x_*), and age-specific net maternity (*l_x_m_x_*) for the laboratory strain (Sfr-WT) and chlorantraniliprole-selected strain (Sfr-SEL-F_6_) of *S. frugiperda* against chlorantraniliprole.

The age-specific survival rate (*l_x_*), female age-specific fecundity (*f_x_*_9_), age-specific fecundity of the total population (*m_x_*), and age-specific net maternity (*l_x_m_x_*) for the laboratory strain (Sfr-WT) and the chlorantraniliprole-selected strain (Sfr-Sel-F_6_) of *S. frugiperda*. The Sfr-WT strain showed higher and more prolonged fecundity, with peak reproduction occurring slightly earlier compared to the selected strain. In contrast, the Sfr-SEL-F_6_ strain exhibited a steeper decline in survival rate and a noticeably reduced fecundity across all metrics (*f_x_*_9_, *m_x_*, and *l_x_m_x_*), indicating that selection pressure from chlorantraniliprole not only reduced overall reproductive output but also delayed and compressed the reproductive window. These findings highlight the potential fitness costs associated with resistance development in *S. frugiperda* (Figure 4).

**Figure 5:**
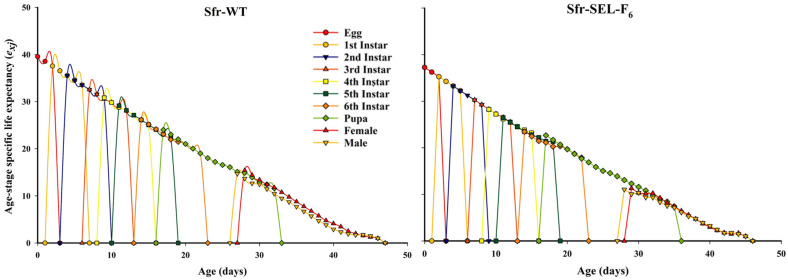
Age-stage specific life expectancy (*e_xj_*) for the laboratory strain (Sfr-WT) and chlorantraniliprole-selected strain (Sfr-SEL-F_6_) of *S. frugiperda* against chlorantraniliprole.

The age-stage specific life expectancy (*e*_xj_) of the laboratory strain (Sfr-WT) and the chlorantraniliprole-selected strain (Sfr-Sel-F_6_) of *S. frugiperda*. In the Sfr-WT strain, individuals at each developmental stage exhibited generally higher life expectancy values, particularly during the early instars and adult stages, indicating better overall survival prospects. Conversely, the Sfr-Sel-F_6_ strain displayed reduced life expectancy across most stages, especially in later larval instars and adult stages. The rapid decline in *e_xj_* among selected individuals suggests that exposure to chlorantraniliprole not only shortened the lifespan but also imposed developmental stress, further supporting the existence of fitness costs associated with resistance selection (Figure 5).

**Figure 6:**
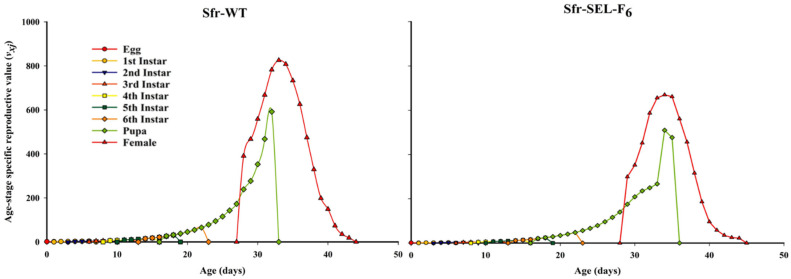
Age-stage specific reproductive value (*v_xj_*) for the laboratory strain (Sfr-WT) and chlorantraniliprole-selected strain (Sfr-SEL-F_6_) of *S. frugiperda* against chlorantraniliprole.

The age-stage specific reproductive value (*v_xj_*) of the laboratory strain (Sfr-WT) and the chlorantraniliprole-selected strain (Sfr-SEL-F_6_) of *S. frugiperda*. The reproductive value represents the future reproductive potential of individuals at a specific age and stage. In the Sfr-WT strain, females reached a peak reproductive value earlier and achieved higher maximum *v_xj_* values compared to the Sfr-SEL-F_6_, indicating a greater and earlier reproductive contribution. In contrast, the Sfr-Sel-F_6_ strain showed a delayed and lower peak in reproductive value, suggesting a reduction in reproductive potential and efficiency. These differences highlight the negative impact of chlorantraniliprole selection on the reproductive fitness of *S. frugiperda*, reinforcing the evidence of fitness costs associated with resistance development (Figure 6).

## 4. Discussion

The present study provides a comprehensive evaluation of the resistance status and fitness consequences associated with chlorantraniliprole selection in *S. frugiperda* field populations from Pakistan. By incorporating baseline toxicity screening, transgenerational selection, realized heritability estimation, and life history comparisons, we offer valuable insights into resistance dynamics and their implications for sustainable pest management strategies.

Our findings revealed significant variation in insecticidal toxicity among seven field-collected *S. frugiperda* populations, with chlorantraniliprole and emamectin benzoate consistently exhibiting high toxicity, whereas lufenuron and bistrifluron demonstrated reduced efficacy. Similar patterns have been documented in other studies, reflecting geographical variation in exposure history and the pest’s capacity to develop differential responses depending on chemical class and selection pressure [54]. A study by Hasnain et al. examined the life history traits of *S. frugiperda* strains with varying susceptibility to chlorantraniliprole, revealing that reduced susceptibility can promote population growth, which is crucial for understanding resistance dynamics and management strategies [55]. Zaidi et al. reported on the resistance of *S. frugiperda* to lambda-cyhalothrin in Pakistan, highlighting the occurrence of cross-resistance and the genetic basis of resistance, which is relevant for understanding the broader implications of resistance management [11]. Guo et al. focused on chlorantraniliprole resistance in *S. frugiperda* in China, emphasizing the need for resistance monitoring and understanding resistance mechanisms, which parallels the findings from Pakistan and underscores the global nature of the issue [56]. Van den Berg and du Plessis discussed the history of insecticide resistance in *S. frugiperda*, noting the role of local selection pressures and the importance of integrated pest management (IPM) strategies [57], which are critical for sustainable pest control. Abbas et al. studied resistance in *Amrasca devastans* to new insecticides in Pakistan, suggesting rotational use of insecticides to manage resistance, a strategy that can be applied to *S. frugiperda* management as well [57,58]. These results underscore the importance of regional resistance monitoring prior to formulating insecticide rotation schemes, particularly for high-risk compounds such as diamides. Studies on different species and geographical areas provide valuable insights into the mechanisms and management of resistance, highlighting the need for comprehensive and adaptive pest management strategies. This broader perspective can help in developing more effective and sustainable approaches to managing resistance in *S. frugiperda* and other agricultural pests.

Following six generations of laboratory selection with chlorantraniliprole, Sfr-SEL-F_6_ exhibited a 4.48-fold increase in LC_50_ compared to the parental F_0_, confirming the ability of *S. frugiperda* to develop resistance under sustained selection pressure. A similar study on cyantraniliprole resistance in *S. frugiperda* showed a 23.97-fold increase in resistance after 13 generations, with a realized heritability of 0.127, suggesting a genetic basis for resistance development [59]. The observed increase in resistance in our study, although moderate, suggests an early warning of resistance evolution, particularly under field conditions where selection pressure is often more variable but persistent. Realized heritability (*h*^2^) is a key metric in understanding how resistance traits are passed through generations. In this study, *h*^2^ of 0.198 further supports the moderate potential for chlorantraniliprole resistance to evolve within populations. Supporting this, Salamat et al. reported a 198.7-fold increase in chlorantraniliprole resistance and a 136.5-fold increase in flubendiamide resistance after 10 generations of selection in *S. frugiperda*, with *h*^2^ values of 0.15 and 0.16, respectively [17]. Their findings also revealed cross-resistance to abamectin and pyriproxyfen, emphasizing the importance of monitoring resistance not only to primary insecticides but also to chemically unrelated compounds [17]. For instance, the realized heritability of cyantraniliprole resistance in *S. frugiperda* was found to be 0.127, indicating a moderate potential for resistance development under selection pressure [59]. In another study, the realized heritability of lambda-cyhalothrin resistance was reported as 0.88, suggesting a high potential for resistance evolution in *S. frugiperda* [11], while heritability of fluxametamide resistance was lower, at 0.084, indicating a slower rate of resistance compared to other insecticides [24]. Furthermore, lambda-cyhalothrin resistance in *S. frugiperda* showed a higher heritability (*h*^2^ = 0.384), indicating a stronger genetic basis and faster spread of resistance alleles under selection [22]. These heritability metrics are crucial for modeling resistance risk and estimating the number of generations required to achieve critical resistance thresholds, such as 10-fold resistance. The moderate heritability and observed resistance development suggest an early warning of resistance evolution, particularly under field conditions where selection pressure is more variable but persistent [22]. Understanding the genetic and biochemical mechanisms of resistance can inform integrated pest management (IPM) strategies to delay resistance development and preserve the efficacy of insecticides [60,61]. Although the observed resistance in *S. frugiperda* to chlorantraniliprole is moderate, it serves as an early indicator of potential resistance evolution under field conditions. Additionally, future study about the role of detoxification enzymes and cross-resistance patterns will underscore the complexity of resistance mechanisms, providing a comprehensive approach to resistance management.

The modeled projections provide important insights into the potential risk of rapid resistance development in *S. frugiperda* under field conditions. Similar to previous findings by Wei et al. who reported that cyantraniliprole resistance increased substantially over 13 generations under continuous selection [59]. Our study predicts that even moderate levels of realized heritability (*h*^2^ = 0.198) can lead to a 10-fold resistance increase in less than 30 generations if selection intensity remains high. Moreover, the strong effect of slope on the generation estimate is consistent with theoretical models emphasizing the role of population heterogeneity in resistance evolution [41]. Likewise, Zaidi et al. demonstrated that higher heritability (*h*^2^ = 0.384) correlated with a faster resistance development to lambda-cyhalothrin in *S. frugiperda*, underscoring the necessity of integrating genetic factors into IRM frameworks [11]. These projections suggest that without rotational use of insecticides and implementation of IPM practices, chlorantraniliprole resistance could escalate quickly under sustained field use. Therefore, understanding the interaction between heritability and selection pressure is essential for refining resistance risk assessment and tailoring localized management strategies.

Beyond the evolution of resistance, our life table analyses clearly highlight the fitness repercussions associated with chlorantraniliprole resistance. The Sfr-Sel-F_6_ strain exhibited notably extended larval and pupal development phases, decreased adult lifespan, shortened oviposition periods, and a significant reduction in fecundity when compared to the susceptible laboratory strain. For instance, in *S. exigua*, the chlorantraniliprole-resistant strains showed increased pre-adult duration and adult longevity but reduced mean fecundity and oviposition days compared to susceptible strain [62]. Similarly, *Plutella xylostella* resistant to lufenuron had a prolonged larval period, with the significant decrease in fecundity. Resistance can lead to lower survival rates at various life stages. For example, in *S. litura*, exposure to chlorpyrifos resulted in reduced larval survival and adult emergence [63]. In *Cnaphalocrocis medinalis*, resistance was associated with fitness costs that could potentially reduce survival rates in the absence of insecticide pressure [64]. These differences led to markedly lower demographic parameters, such as the intrinsic rate of increase (*r*), net reproductive rate (*R*_0_), and finite rate of increase (*λ*), with a relative fitness (*R_f_*) of 0.61 which is consistent with the findings of Roy et al. who observed reduced reproductive success and survivorship in fluxametamide-resistant strains [24]. The fitness costs associated with resistance suggest that in the absence of selection pressure, resistant alleles may be outcompeted by susceptible ones, leading to a potential reversion of resistance [65], as reported in *S. exigua*, where fitness costs disappeared when insecticide pressure was removed [62]. Additionally, the persistence of resistance in natural populations can be influenced by factors such as migration and mating competitiveness, which may not always be accounted for in laboratory studies [65]. Therefore, future studies should focus on understanding the molecular biology underlying resistance mechanisms that can provide deeper insights into these dynamics and inform more effective pest management strategies.

The evolution of insecticide resistance in *S. frugiperda* is often accompanied by substantial fitness costs that manifest in key life history traits. In our study, the chlorantraniliprole-selected strain (Sfr-Sel-F_6_) exhibited significantly reduced population growth metrics compared to the unselected wild-type (Sfr-WT), including lower intrinsic (*r*) and finite (*λ*) rates of increase, a sharp decline in net reproductive rate (*R*_0_), and a 39% reduction in relative fitness. These demographic shifts were further supported by delayed larval development, prolonged pupation, reduced survival rates, and compressed reproductive peaks, indicating that resistance development compromises overall biological performance. Findings from other studies reinforce the impact of sublethal and transgenerational insecticide exposure on *S. frugiperda* population dynamics. Sublethal doses of emamectin benzoate and spinetoram have been shown to extend developmental duration, reduce fecundity, and impair survival, leading to suppressed population growth [66]. Similarly, cyantraniliprole exposure has demonstrated transgenerational effects, with altered reproductive traits and reduced fecundity in subsequent generations [53]. Furthermore, a recent study by Lv et al. demonstrated that chlorantraniliprole at sublethal doses significantly affects the development and reproductive traits of *S. frugiperda* [67].

In conclusion, this study reveals that *S. frugiperda* populations in Pakistan are in the early stages of developing resistance to chlorantraniliprole, as indicated by moderate realized heritability and measurable fitness costs. These findings underscore the importance of resistance monitoring and transgenerational selection studies to inform region-specific, data-driven insecticide resistance management (IRM) strategies. Moderate heritability values suggest that resistance traits can increase under continued selection pressure [20,24,56]. Associated fitness costs—such as reduced fecundity, prolonged development, and lower survival—can be strategically exploited through insecticide rotation and mode-of-action diversification to suppress resistant individuals [18,55,68]. Hence, regular surveillance and adaptive IRM approaches are essential to detect early resistance shifts and adjust control tactics accordingly.

Although this study focuses on *S. frugiperda* in Pakistan, its findings have broader relevance for managing insecticide resistance in other regions and pest species. The principles of rotating insecticides, monitoring resistance, and leveraging fitness costs are widely applicable across diverse agroecological contexts. However, resistance dynamics can vary significantly depending on species and environmental conditions. Therefore, IRM strategies should remain adaptable and guided by ongoing research and field data to ensure long-term effectiveness.

## Figures and Tables

**Table 1:** Insecticidal toxicity of different insecticides against the field populations of *S. frugiperda* from Pakistan.

Insecticide	Locations	LC_50_ (95% FL)	Slope	* *χ*^2^ (*df*)	*p*-Value	R^2^ Value
Bistrifluron	CT	3.923 (2.704–5.564)	1.491 ± 0.207	20.831 (13)	0.076	0.846
	SC	2.410 (1.590–3.491)	1.385 ± 0.180	13.215 (13)	0.43	0.899
	AR	2.314 (1.558–3.252)	1.582 ± 0.204	11.556 (13)	0.564	0.879
	GJ	3.118 (2.123–4.400)	1.515 ± 0.203	15.041 (13)	0.304	0.891
	NL	2.239 (1.530–3.096)	1.738 ± 0.221	12.035 (13)	0.525	0.891
	TTS	3.455 (1.763–5.973)	1.751 ± 0.232	31.372 (13)	0.002	0.785
	SHW	1.238 (0.738–1.920)	1.143 ± 0.149	12.608 (13)	0.478	0.859
Chlorantraniliprole	CT	1.143 (0.859–1.450)	2.077 ± 0.299	6.847 (13)	0.873	0.91
	SC	1.229 (0.965–1.521)	2.388 ± 0.321	3.884 (13)	0.946	0.99
	AR	1.240 (0.977–1.530)	2.434 ± 0.324	4.483 (13)	0.925	0.98
	GJ	1.274 (0.994–1.586)	2.288 ± 0.310	4.867 (13)	0.929	0.98
	NL	1.490 (1.210–1.817)	2.651 ± 0.334	3.972 (13)	0.95	0.99
	TTS	0.983 (0.742–1.232)	2.293 ± 0.329	3.965 (13)	0.93	0.99
	SHW	1.341 (1.034–1.688)	2.128 ± 0.294	4.907 (13)	0.948	0.98
Flubendiamide	CT	1.728 (0.991–2.778)	0.998 ± 0.144	11.088 (13)	0.603	0.911
	SC	2.367 (1.576–3.443)	1.370 ± 0.167	9.009 (13)	0.772	0.931
	AR	1.560 (0.948–2.395)	1.152 ± 0.152	7.877 (13)	0.851	0.93
	GJ	2.867 (1.969–4.061)	1.519 ± 0.182	8.351 (13)	0.820	0.957
	NL	2.164 (1.432–3.140)	1.387 ± 0.169	9.117 (13)	0.764	0.942
	TTS	4.678 (3.248–6.683)	1.473 ± 0.188	15.791 (13)	0.260	0.914
	SHW	0.919 (0.512–1.458)	1.115 ± 0.154	6.413 (13)	0.929	0.926
Lufenuron	CT	4.402 (1.805–7.864)	1.420 ± 0.211	29.763 (13)	0.005	0.692
	SC	7.973 (6.709–9.159)	4.176 ± 0.611	7.465 (13)	0.876	0.878
	AR	3.610 (2.163–5.414)	1.189 ± 0.178	16.904 (13)	0.203	0.902
	GJ	2.791 (1.645–4.208)	1.219 ± 0.172	18.86 (13)	0.127	0.819
	NL	4.031 (1.724–7.001)	1.443 ± 0.210	27.302 (13)	0.011	0.722
	TTS	2.087 (1.182–3.217)	1.200 ± 0.165	18.592 (13)	0.136	0.812
	SHW	4.276 (1.748–7.068)	1.736 ± 0.263	29.444 (13)	0.005	0.743
Emamectin benzoate	CT	0.301 (0.159–0.495)	1.005 ± 0.147	9.252 (13)	0.753	0.855
	SC	0.166 (0.069–0.300)	0.897 ± 0.145	7.104 (13)	0.896	0.867
	AR	0.323 (0.181–0.514)	1.095 ± 0.153	6.998 (13)	0.902	0.911
	GJ	0.199 (0.098–0.333)	1.045 ± 0.154	5.154 (13)	0.971	0.924
	NL	0.250 (0.133–0.403)	1.096 ± 0.155	5.914 (13)	0.949	0.916
	TTS	0.553 (0.343–0.838)	1.204 ± 0.159	8.081 (13)	0.838	0.918
	SHW	0.302 (0.171–0.476)	1.133 ± 0.157	8.317 (13)	0.822	0.904
Indoxacarb	CT	3.493 (2.329–4.755)	1.629 ± 0.236	9.582 (13)	0.727	0.866
	SC	4.087 (2.976–5.291)	2.024 ± 0.263	10.874 (13)	0.621	0.876
	AR	4.690 (3.370–6.152)	1.814 ± 0.250	10.911 (13)	0.618	0.886
	GJ	4.247 (3.050–5.561)	1.867 ± 0.251	12.075 (13)	0.521	0.842
	NL	5.021 (3.771–6.374)	2.167 ± 0.281	8.776 (13)	0.789	0.92
	TTS	4.412 (3.277–5.638)	2.151 ± 0.275	9.045 (13)	0.769	0.904
	SHW	5.044 (3.697–6.535)	1.914 ± 0.259	8.991 (13)	0.773	0.927
Cyantraniliprole	CT	1.126 (0.568–1.928)	0.868 ± 0.136	6.571 (13)	0.922	0.906
	SC	0.415 (0.166–0.770)	0.851 ± 0.136	8.363 (13)	0.819	0.893
	AR	0.515 (0.280–0.828)	1.166 ± 0.153	8.801 (13)	0.787	0.907
	GJ	0.801 (0.393–1.378)	0.891 ± 0.136	11.319 (13)	0.584	0.895
	NL	0.979 (0.570–1.525)	1.149 ± 0.150	10.79 (13)	0.628	0.896
	TTS	0.922 (0.532–1.446)	1.134 ± 0.148	9.074 (13)	0.767	0.916
	SHW	0.781 (0.413–1.282)	1.013 ± 0.142	8.853 (13)	0.783	0.916

* Chi square value (*χ*^2^), fiducial limits (FL) and degrees of freedom (*df*) are calculated by Probit analysis using SPSS v.17.0 software (SPSS Inc., Chicago, IL, USA).

**Table 2:** Transgenerational selection of the field collected population of *S. frugiperda* (NL with relatively higher LC_50_ and had good culture in the laboratory conditions) after continuous selection using LC_70_ of each subsequent generation.

Generation	LC_50_ (95% FL) mg/L	Slope	*χ*^2^ (*df*)	R^2^ Value	*p*-Value	* RR
F_0_	1.217 (0.945–1.513)	2.275 ± 0.310	6.487 (13)	0.922	0.926	1.0
F_1_	2.134 (1.705–2.567)	2.743 ± 0.367	5.892 (13)	0.942	0.949	1.75
F_2_	3.958 (3.306–4.578)	3.369 ± 0.473	7.573 (13)	0.944	0.870	3.25
F_3_	4.358 (3.785–4.925)	4.175 ± 0.529	12.192 (13)	0.867	0.512	3.58
F_4_	4.537 (3.967–5.098)	4.445 ± 0.563	10.378 (13)	0.896	0.662	3.73
F_5_	4.873 (4.278–5.483)	4.307 ± 0.546	12.401 (13)	0.88	0.495	4.00
F_6_	5.452 (4.832–6.099)	4.561 ± 0.601	11.076 (13)	0.901	0.604	4.48

* Resistance ratio (RR) = LC_50_ of subsequent generation (F_n_)/LC_50_ of the parental generation (F_0_).

**Table 3:** Realized heritability (*h*^2^) for resistance against chlorantraniliprole in *S. frugiperda*.

Generations (n)	Estimation of Selection Response (*R*)					Estimation of Selection Difference (*S*)			*h* ^2^
10 (F_0_–F_6_)	Initial LC_50_	Final LC_50_	*R*	*p*	*i*	Mean slope	*σ_P_*	*S*	0.198
	1.217	5.452	0.109	6.42	2.029	3.696	0.271	0.549	

*R*, Selection response; *p*, Survival percentage; *i*, Intensity of selection; *σ_P_*; Phenotypic variance; *S*, Selection differential; *h*^2^, Realized heritability.

**Table 4:** Duration of various developmental stages of the chlorantraniliprole-selected strain (Sfr-Sel-F_6_) of *S. frugiperda* and the laboratory strain (Sfr-WT).

Parameters	Sfr-WT	Sfr-Sel-F_6_	*p*-Value	F-Value	*df*
Eggs (d)	2.89 ± 0.03 a	2.93 ± 0.03 a	0.325	0.972	1, 198
1st Instar (d)	2.63 ± 0.05 a	2.59 ± 0.05 a	0.572	0.320	1, 198
2nd Instar (d)	2.43 ± 0.04 b	2.57 ± 0.05 a	0.048	3.958	1, 198
3rd Instar (d)	2.76 ± 0.06 a	2.70 ± 0.05 a	0.417	0.661	1, 197
4th Instar (d)	2.7 ± 0.05 a	2.75 ± 0.06 a	0.587	0.296	1, 195
5th Instar (d)	2.81 ± 0.06 a	2.78 ± 0.05 a	0.680	0.170	1, 194
6th Instar (d)	3.20 ± 0.06 b	3.62 ± 0.07 a	<0.001	21.625	1, 181
Total larval duration (d)	16.54 ± 0.13 b	17.05 ± 0.13 a	0.006	7.782	1, 180
Male pupal duration (d)	10.50 ± 0.14 b	11.28 ± 0.18 a	0.001	11.540	1, 74
Female pupal duration (d)	11.06 ± 0.13 b	11.51 ± 0.13 a	0.016	6.004	1, 86
Male adult (d)	12.36 ± 0.43 a	9.35 ± 0.49 b	<0.001	21.138	1, 74
Female adult (d)	12.87 ± 0.28 a	9.85 ± 0.48 b	<0.001	30.734	1, 86

Note: Values are shown as mean ± standard error. Different lower-case letters in a row indicate significant differences using Tukey’s multiple comparison test (*p* < 0.05).

**Table 5:** The life table parameters of the chlorantraniliprole-selected strain (Sfr-Sel-F_6_) of *S. frugiperda* and the laboratory strain (Sfr-WT).

Parameters	Sfr-WT	Sfr-Sel-F_6_	*p*-Value	F-Value	*df*
Male mean longevity (d)	42.11 ± 0.50 a	40.60 ± 0.65 a	0.073	3.310	1, 74
Female mean longevity (d)	43.51 ± 0.37 b	41.46 ± 0.55 a	0.002	9.966	1, 86
APOP (d)	2.87 ± 0.05 a	2.81 ± 0.07 a	0.437	0.610	1, 80
TPOP (d)	33.52 ± 0.23 b	34.44 ± 0.30 a	0.016	6.023	1, 80
Mean fecundity	1486.48 ± 38.02 a	1153.17 ± 25.34 b	<0.001	47.220	1, 80
Oviposition duration (d)	5.41 ± 0.07 a	5.14 ± 0.08 b	0.015	6.241	1, 80

Note: Values are shown as mean ± standard error. Different lower-case letters in a row indicate significant differences using Tukey’s multiple comparison test (*p* < 0.05).

**Table 6:** The demographic parameters of the chlorantraniliprole-selected strain (Sfr-Sel-F_6_) of *S. frugiperda* and the laboratory strain (Sfr-WT).

Parameters	Sfr-WT	Sfr-Sel-F_6_	*p*-Value
Intrinsic rate of increase (*r*) (d^−1^)	0.1789 ± 0.003 a	0.1627 ± 0.004 b	0.002
Finite rate of increase (*λ*) (d^−1^)	1.1960 ± 0.004 a	1.1767 ± 0.005 b	0.002
Net reproduction rate (*R*_0_) (offspring/individual)	684.116 ± 76.01 a	415.517 ± 56.16 b	0.005
Mean generation time (*T*) (d)	36.479 ± 0.242 a	37.068 ± 0.282 a	0.112
Female ratio (*N_f_*/*N*)	0.47 ± 0.05 a	0.41 ± 0.05 a	0.430
Relative fitness (*R_f_*)	-	0.61	-

Note: The mean ± standard error values were estimated using the paired bootstrap test in the age-stage TWOSEX-MS chart program with 100,000 bootstrap resamplings [48,49]. Different lower-case letters indicate a significant difference (*p* < 0.05). *R_f_* = R_0_ of Sfr-Sel-F_6_/R_0_ of Sfr-WT [53].

## Data Availability

The original contributions presented in this study are included in the article. Further inquiries can be directed to the corresponding author.
